# Effects of vitamin D supplementation on fasting glucose, dyslipidemia, blood pressure, and abdominal obesity among patients with metabolic syndrome: a protocol for systematic review and meta-analysis of randomized controlled trials

**DOI:** 10.1186/s13643-020-01433-3

**Published:** 2020-08-11

**Authors:** Séphora Louyse Silva Aquino, Aline Tuane Oliveira Cunha, Josivan Gomes Lima, Karine Cavalcanti Maurício Sena-Evangelista, Antonio Gouveia Oliveira, Ricardo Ney Cobucci, Lucia Fatima Campos Pedrosa

**Affiliations:** 1 Postgraduate Program in Health Sciences, Federal University of Rio Grande do Norte, Natal, RN 59078-970 Brazil; 2 Collaborative Researcher in Postgraduate Program in Nutrition, Federal University of Rio Grande do Norte, Av. Senador Salgado Filho, 3000-Lagoa Nova, Natal, CEP 59078970 Brazil; 3 Department of Clinical Medicine, Endocrine Unit, Federal University of Rio Grande do Norte, Natal, RN 59010-180 Brazil; 4 Department of Nutrition, Federal University of Rio Grande do Norte, Av.Sen. Salgado Filho, n° 3000, Lagoa Nova, Natal, RN CEP 59078-970 Brazil; 5 Department of Pharmacy, Federal University of Rio Grande do Norte, Natal, RN 59012570 Brazil; 6 Postgraduate Program in Biotechnology and Medicine School, Potiguar University-UnP, Natal, RN 59056-000 Brazil

**Keywords:** Metabolic syndrome, Vitamin D supplementation, Systematic review, Randomized controlled trials

## Abstract

**Background:**

Vitamin D deficiency can play a role in extraskeletal functions that are involved with a set of risk factors associated with metabolic syndrome (MetS). The purpose of this review is to investigate the impact of vitamin D supplementation on fasting glucose, dyslipidemia, blood pressure, and abdominal obesity among patients with MetS.

**Methods:**

EMBASE, Medline, Web of Science, Lilacs, the Cochrane Central Register of Controlled Trials, clinicaltrials.gov databases, and grey literature will be systematically searched for randomized controlled trials (RCTs) of vitamin D supplementation compared with placebo, through December 2020. We will include in the study patients with MetS diagnosed by the criteria set forth by the National Cholesterol Education Program Adult Treatment Panel III or the International Diabetes Federation. The effect of oral vitamin D supplementation on lipid profile improvement (triglycerides, high-density lipoprotein cholesterol—HDL-C) is this review’s primary outcome. The systematic review will be performed following the Preferred Reporting Items for Systematic Reviews and Meta-Analyses (PRISMA) guidelines. The study screening, data extraction, and quality assessment will be fulfilled by two independent reviewers according to the Cochrane Risk of Bias tool (RoB 2.0). The results of the systematic review will be provided according to the type of intervention, characteristics of the target population, the methods of measurement of vitamin D, the calculated vitamin D concentrations, types of biological samples, and types of outcomes. Meta-analyses will be conducted where appropriate. The Cochran’s *Q* test and the *I*^2^-heterogeneity test will be used to assess the presence of heterogeneity and whether the fixed or the random-effects model would be appropriate for combining study results using the inverse variance method or the DerSimonian-Lair method, respectively. Publication bias will be evaluated using funnel plots and Egger’s and Begg’s tests. The strength of the evidence will be assessed according to the Grading of Recommendations Assessment, Development, and Evaluation (GRADE).

**Discussion:**

This systematic review will assess the effects of vitamin D supplementation on fasting glucose and triglyceride levels, waist circumference and mean blood pressure, and HDL-C among individuals with MetS. These findings may assist with decision-making within a clinical setting.

**Systematic review registration:**

PROSPERO registration number CRD42019123212

## Background

Metabolic syndrome (MetS) is a complex disorder characterized by a set of cardiometabolic risk factors, including hyperglycemia, hypertriglyceridemia, low levels of high-density lipoprotein cholesterol (HDL-C), increased waist circumference, and high blood pressure [1].

Vitamin D deficiency is a global health problem associated with rickets and growth retardation in children, and osteoporosis and osteomalacia in adults. In addition, in recent years, there has been increasing evidence to demonstrate the extraskeletal role of vitamin D. Vitamin D deficiency has also been linked to many acute and chronic illnesses including some cancers, autoimmune diseases, cardiovascular disease, type 1 and type 2 diabetes mellitus, infectious diseases, and neurocognitive dysfunction, among others [2].

Previous research points to the worldwide prevalence of 25-hydroxyvitamin D [25(OH)D] deficiency, even in countries with high ultraviolet radiation (UVR), due to low exposure to UVB radiation, low dietary intake of vitamin D, and metabolic alterations [3]. In relation to these findings in sunny countries, a meta-analysis of seventy-two studies showed the prevalence of vitamin D deficiency and insufficiency to be 28.16% and 45.26%, respectively, for the Brazilian population [4].

In this regard, the cardiometabolic factors in individuals with MetS may interfere with the metabolic activation pathways of vitamin D, thus triggering vitamin D deficiency. However, a supply of vitamin D through diet or exposure to sun does not appear to be enough in individuals with MetS [5].

Observational studies have shown a negative association between low serum concentrations of [25(OH)D] and components of MetS, such as fasting glycemia, arterial hypertension, and triglycerides [6–8]. Conversely, a few studies have demonstrated that vitamin D supplementation was beneficial in decreasing serum triglycerides, waist circumference, insulin resistance, and biomarkers of cardiometabolic risk in healthy adult individuals and patients with MetS [9–11]. Nevertheless, it is uncertain whether supplementation with vitamin D is effective in improving components of MetS because of the controversiality of results [12–14].

In general, discrepancies in studies can be attributed to differences in doses, types of vitamin D supplements used, and duration of the intervention. To the best of our knowledge, the vitamin D dose administered in the studies varied per day, week, month, or year, and also consisted of a large range (700 IU to 8500 IU) per day. Additionally, the duration of intervention was between 2 and 12 months, and the studies were carried out in a variety of different geographical locations and regions [10–12, 14–19].

These uncertainties regarding the efficacy of vitamin D supplementation on the components of MetS make clinical decision-making more difficult. Therefore, it is relevant to develop this systematic review and meta-analysis to provide the best, most up-to-date evidence upon which to base clinical decisions related to vitamin D supplementation in patients with MetS.

## Aims and hypothesis

The overall purpose of this systematic review is to evaluate the efficacy of oral vitamin D supplementation in individuals with MetS in randomized controlled trials (RCTs). The primary aim is to assess the effect of oral vitamin D supplementation versus placebo by decreasing serum concentration of triglycerides and increasing HDL-C. The secondary aim is to assess the effect of oral vitamin D supplementation by decreasing serum concentration of fasting glucose and mean blood pressure, and reducing waist circumference and mortality from cardiovascular events.

Thus, the hypothesis of this systematic review is “Does vitamin D supplementation reduce blood glucose, dyslipidemia, mean blood pressure, and abdominal obesity in subjects with MetS?”.

## Methods/design

### Type of studies

To be eligible for inclusion, studies must be randomized controlled trials (RCTs) of vitamin D supplementation with intervention compared to a placebo in adult individuals diagnosed with MetS.

### Eligibility criteria

Selection criteria using the PICO (Population, Intervention, Comparison, Outcomes) framework is shown in Table 1.

**Table 1:** PICO for study inclusion

Participants (P)	Intervention (I)	Comparison (C)	Outcomes (O)
**Inclusion criteria**			
Participants with MetS diagnosed by NCEP-ATP III (1), or IDF (2) criteria, aged ≥ 18 years, and with inadequate vitamin D status	Vitamin D supplementation in the form of vitamin D3 (cholecalciferol) or vitamin D2 (ergocalciferol) at any dose, given orally, daily, weekly, or monthly	RCTs with intervention compared to placebo	Fasting glucose levels (mg/dL), triglyceride levels (mg/dL), waist circumference (cm), mean blood pressure (mmHg), and HDL- C (mg/dL) (two or more outcomes will be considered in subgroup analyses)
**Exclusion criteria**			
Participants with MetS aged under 18 years	Vitamin D supplements with other vitamin and chemical element supplements; vitamin D supplementation in fortified foods as the amount of vitamin cannot be defined accurately; studies that used active vitamin D supplementation (1.25-dihydroxy vitamin D)		

*MetS* metabolic syndrome, *NCEP-ATPIII* National Cholesterol Education Program Adult Treatment Panel III, *IDF* International Diabetes Federation, *RCTs* randomized controlled trials adapted from Mousa et al. [20]

### Type of outcome measures

#### Primary outcome

Our primary outcome will be the effect of oral vitamin D supplementation on the improvement of lipid profile (triglycerides, HDL-C).

#### Secondary outcome

The secondary outcome includes improvement of fasting glucose, mean blood pressure, and waist circumference (continuous outcome), the proportion of patients with symptomatic improvement according to the number of MetS components, compared to placebo, and reduction of mortality from cardiovascular events.

### Search strategy

A search will be conducted using a combination of free-text and medical subject heading (MeSH) search terms, text words, and keywords based on each database characteristic focusing on synonyms of MeSH and vitamin D supplementation. Key search terms (both MESH and keyword terms) will include the following: metabolic syndrome, vitamin D supplementation, vitamin D3 (cholecalciferol) supplementation, vitamin D2 (ergocalciferol) supplementation, and randomized clinical trials. Our search strategy will be adapted for searches in the other databases to be included in this review.

The following databases will be searched for articles published through 2020, with no restrictions on language: EMBASE, Medline, Web of Science, Lilacs, the Cochrane Central Register of Controlled Trials, clinicaltrials.gov, and grey literature (Google Scholar). References will be manually reviewed to identify supplementary studies of interest. The systematic review will be performed following the Preferred Reporting Items for Systematic Reviews and Meta-Analyses (PRISMA) guidelines [21]. Corresponding authors of any abstract of interest will be contacted for further study details.

### Selection of studies

Two authors (SLSA and ATOC) will independently screen the search results. Any disagreements will be resolved by consensus. Where consensus is not reached, a third reviewer (KCMS) will be consulted in order to reach a decision. The Rayyan QCRI web tool will be used to manage the selection process [22].

For trials that include duplicate data, we will consider some of the most useful criteria for comparing reports, including (1) trial identification numbers (e.g., ClinicalTrials.gov Identifier (NCT number)); ISRCTN; Universal Trial Number (UTN) (assigned by the ICTRP); other identifiers such as those from the sponsor; (2) author names; (3) location and setting; (4) specific details of the interventions (e.g., dose and frequency); (5) number of participants and baseline data; and (6) date and duration of the study. Then, if duplicated data is identified, we will consider only the latest published paper [23].

### Data collection and analysis

Data will be carefully extracted from all eligible publications, including first authors’ last names, year of publication, place of the study (country), study design, primary objective, population, sample size, follow-up period, inclusion/exclusion criteria, types of measurement methods of vitamin D, vitamin D concentration, type of biological sample, type of vitamin D supplement, type of control used, and primary results.

For binary outcomes, the analysis will be by intention-to-treat, and data to be collected will include the number of randomized patients in each trial arm and the number with successful outcomes. For interval variables, the data to be collected will be the mean and standard deviation of the study variables in each trial arm. If only point estimates of 95% confidence intervals for the difference between trial arms are reported, standard deviation may be obtained only in trials with equal sample sizes. This will be achieved by dividing the absolute value of difference of the point estimate to one of the confidence limits by the product of the square root of 2 divided by the common sample size “n” and the *T* value of the Student’s *t* distribution with 2n-2 degrees of freedom exceeded in both directions, with a probability of 0.05. Clinical trials that compare values at the last observation, trials that compare the change from baseline, and trials that present only between-group differences will be analyzed separately.

### Quality assessment

Three reviewers, SLSA, RNC, and LFCP, will independently assess the risk of bias in included studies using the guidelines of Cochrane Handbook for Systematic Reviews of Interventions version 6.0 (Rob 2) [24]. This tool comprises five bias domains: (1) bias arising from the randomization process, (2) bias due to deviation from intended interventions, (3) bias due to missing outcome data, (4) bias in measurement of the outcome, and (5) bias in selection of the reported result. The domains were selected to address all important mechanisms by which bias can be introduced into the results of a trial, based on a combination of empirical evidence and theoretical considerations. The response options for an overall risk-of-bias judgment are the same as for individual domains: low risk of bias, with some concerns or high risk of bias.

### Data synthesis

This systematic review will be carried out using Review Manager (RevMan 5.3) and Stata 15 (Stata Corp., College Station, TX, USA). Using a data extraction form, the following data will be obtained: methods of measurement of vitamin D, vitamin D concentration, type of biological sample, type of intervention (dose, duration, and frequency), characteristics of the target population, and outcomes. After extracting the data, the authors will determine whether a meta-analysis is possible. It is expected that as every outcome is measured in the same units across studies, the measure of effect size for all outcomes will be the difference in means; if not, the standardized mean difference will be adopted as a measure of effect size.

The 95% confidence intervals of effect sizes will be constructed according to their type and the selected analytical model. Therefore, if the fixed effects model is adopted, the standard error of effect sizes defined as difference in means or standardized mean difference will be estimated using the inverse variance method; for risk difference, the Mantel-Haenszel method will be used. When the random effects model is selected, the standard error of all effect sizes will be estimated using the DerSimonian Method [25].

### Assessment of heterogeneity

The presence of heterogeneity will be tested with Cochrane’s *Q* test for each outcome variable, and its magnitude will be estimated with the *I*^2^ statistic, a measure of heterogeneity across studies. Meta-analytic estimates of the effect size and confidence interval will be obtained from the random effects model, unless there are reasons to believe the fixed effects model may be adequate. These include evidence of a very small degree of heterogeneity based on a non-significant *Q* test plus an *I*^2^ statistic less than 50%, if there is a small number (< 10) of RCTs, or if the number of RCTs is greater, the former plus an evaluation of the similarity across studies regarding eligibility criteria, study populations, trial designs, duration, doses administered, and outcomes assessment.

### Assessment of publication biases

Publication bias will be assessed visually with funnel plots, as well as with Egger’s and Begg’s tests for studies included in the review.

### Quality of evidence

The strength of the evidence will be evaluated according to the Grading of Recommendations Assessment, Development, and Evaluation (GRADE) using the GRADE PRO software (https://gdt.gradepro.org). The GRADE is an instrument used to assess the quality of evidence at 4 levels: high, moderate, low, and very low, and to analyze each of the following domains: randomization process, missing outcome data, measurement of the outcome, and selection of the reported result. RNC and LFCP will be used to evaluate the quality of evidence using GRADE, and KCMS will check and resolve discrepancies.

### Subgroup analysis

Subgroup analyses will be conducted if a sufficient number of studies are available to determine which of the following modifiers explain the heterogeneity: baseline vitamin D status, vitamin D dose, duration and frequency of the administration, form of vitamin D supplementation, and location (country) of supplementation.

## Discussion

Observational studies assessing the association between vitamin D status in patients with MetS and predictive factors, as well as RCTs carried out with vitamin D supplementation, have shown controversial results. In both types of studies, there is no evidence that the lower levels of vitamin D observed in patients with MetS were a cause or a consequence of MetS, or if vitamin D supplementation had benefit across all components of MetS [12, 14, 16, 26].

Most studies that assess vitamin D supplementation consider isolated cardiometabolic factors of MetS. However, this systematic review will assess whether vitamin D supplementation is beneficial to individuals with MetS, and the findings may help in clinical practice decisions related to the dose, duration, and frequency of vitamin D supplementation. To the best of our knowledge, this review will be the first to evaluate vitamin D supplementation in the context of all the components of MetS.

## Data Availability

Not applicable.

## Abbreviations

MetS: Metabolic syndrome
HDL-C: High-density lipoprotein cholesterol
PRISMA: Preferred Reporting Items for Systematic Reviews and Meta-Analyses
GRADE: Grading of Recommendations Assessment, Development, and Evaluation
UVR: Ultraviolet radiation
RCTs: Randomized controlled trials
PICO: Population, Intervention, Comparison, and Outcomes
NCEP-ATPIII: National Cholesterol Education Program Adult Treatment Panel III
IDF: International Diabetes Federation
MeSH: Medical Subject Headings
